# Antibacterial Resin Composites with Sustained Chlorhexidine Release: One-Year In Vitro Study

**DOI:** 10.3390/pharmaceutics17091144

**Published:** 2025-09-01

**Authors:** Flávia Gonçalves, Larissa Sampaio Tavares Silva, Julia Noborikawa Roschel, Greca de Souza, Luiza de Paiva Mello Campos, Gustavo Henrique Varca, Duclerc Parra, Mirko Ayala Perez, Antonio Carlos Gordilho, William Cunha Brandt, Leticia Boaro

**Affiliations:** 1 Faculdade de Odontologia, Universidade Santo Amaro, Av. Prof. Eneas de Siqueira Neto, 340, Sao Paulo 04829-300, SP, Brazil; flgoncalves@prof.unisa.br (F.G.); junoborikawa@hotmail.com (J.N.R.); jgreca@estudante.unisa.br (G.d.S.); ayalamirko@gmail.com (M.A.P.); gordilho.a@gmail.com (A.C.G.); williambrandt@yahoo.com.br (W.C.B.); 2 Departamento de Biomateriais e Biologia Oral, Faculdade de Odontologia, Universidade de São Paulo, Av. Prof. Lineu Prestes, 2222, Sao Paulo 05508-000, SP, Brazil; larissatsampaio@gmail.com; 3 Instituto de Pesquisas Energéticas e Nucleares IPEN-CNEN/SP, Av. Prof. Lineu Prestes, 2242, Sao Paulo 05508-000, SP, Brazil; luizamellocampos@gmail.com (L.d.P.M.C.); gustavovarca@hotmail.com (G.H.V.); dfparra@ipen.br (D.P.); 4 College of Dentistry, University of Saskatchewan, 105 Wiggins Rd., Saskatoon, SK S7N 5E5, Canada

**Keywords:** resin composites, antimicrobial, chlorhexidine, long-term release

## Abstract

**Background:** The addition of chlorhexidine in dental restorative materials is a promising strategy to reduce the recurrence of tooth decay lesions. However, the main challenge is to develop materials with antimicrobial activity in the long term. **Objective:** This study analyses the effect of filler type and concentration of resin composites supplemented with chlorhexidine loaded in carrier montmorillonite particles (MMT/CHX) regarding their chemical, physical, and short- and long-term antimicrobial proprieties. **Materials:** Experimental composites were synthesized with 0, 30, or 60% filler in two ratios, 70/30 and 80/20, of barium glass/colloidal silica, respectively, and 5 wt% MMT/CHX. Conversion was measured using near Fourier-transform infrared spectrometry. Sorption and solubility were determined by specimen weight before and after drying and immersing in water. Flexural strength (FS) and elastic modulus (E) were determined by three bending tests using a universal test machine. Chlorhexidine release was monitored for 50 days. *Streptococcus mutans* UA159 was used in all microbiological assays. Inhibition halo assay was performed for 12 months and, also, biofilm growth for the specimens and colony-forming unit (CFU). Remineralization assay was used on restored teeth using measurements of microhardness Knoop and CFUs. **Results:** Conversion, sorption, and solubility were not affected by filler type and concentration. FS and E increase with the filler concentration, independent from filler type. Chlorhexidine was significantly released for 15 days for all experimental materials, and the increase in filler concentration decreased its release. Halo inhibition was observed for a longer time (12 months) in materials with 60 wt% filler at 70/30 proportion. Also, 60 wt% filler materials, independent from the filler ratio, reduced the CFU in relation to the control group from 8 to 12 months. In the remineralization assay, besides the absence of differences in hardness among the groups, after biofilm growth, the CFU was also significantly lower in materials with 60 wt% filler. **Conclusions:** Materials with 60% filler, preferentially with 70% barium glass and 30% silica, and 5% MMT/CHX particles demonstrated long-term antimicrobial activity, reaching 12 months of effectiveness. Also, this formulation was associated with higher mechanical properties and similar conversion, sorption, and solubility compared to the other materials.

## 1. Introduction

Composite resins stand out as the preferred materials for direct restoration due to their commendable aesthetic and mechanical properties. One of the primary reasons for failures and replacements in composite resin restorations is the emergence of caries lesions near the restoration site [1,2]. While maintaining proper oral hygiene and adhering to a non-cariogenic diet are pivotal measures to avert these lesions, the incorporation of antimicrobial restorative materials holds promise as a potential complement to patient treatment [3].

Numerous antimicrobial restorative materials have been proposed in the literature [1,2,3]. Chlorhexidine (CHX) has emerged as a promising agent for several reasons: its established efficacy in preventing and treating oral infections [4], its role in enhancing the longevity of restorations through the inhibition of metalloproteinases [5], and its effectiveness as a mouthwash [4], as well as its application in combination with resin composites [2,6].

A study formulated composites loaded with CHX and observed that direct incorporation into the composite resulted in increased surface roughness, diminished mechanical properties, and a rapid, excessive release of CHX [6]. Conversely, when CHX was associated with a carrier particle, such as porous silica, the material’s properties were maintained, and the release exhibited a more gradual and prolonged profile, in addition to inhibiting the growth of *S. mutans* and *L. casei* [6].

The montmorillonite (MMT) may serve as a viable alternative for CHX delivery due to its capacity to absorb organic molecules and facilitate controlled drug release [7,8]. This is achieved through its structure consisting of interleaved lamellar layers, featuring a central octahedral alumina structure between two layers of tetrahedral silica structure [9].

A prior study noted that the incorporation of the MMT/CHX complex into experimental composites did not alter the degree of conversion of dimethacrylates [10]. Additionally, at a concentration of 5%, the MMT/CHX complex demonstrated antimicrobial activity against *S. mutans*, *P. gingivalis*, and *S. aureus* without compromising the mechanical properties and the degree of conversion [2]. Despite the significant potential of this material and promising short-term effects, the addition of different inorganic fillers may alter the release and antimicrobial effect of the material through both physical and chemical interactions with MMT and CHX molecules.

Therefore, for potential clinical application, it is necessary to conduct studies on the conjugation of MMT/CHX with various concentrations and types of filler particles. Additionally, an investigation into the short- and long-term antimicrobial properties of this material is crucial.

The aim of this study is to assess the immediate and long-term antimicrobial properties and evaluate the physicochemical properties of experimental composites loaded with 5% MMT/CHX in conjunction with various concentrations and ratios of barium glass and colloidal silica. The null hypothesis posits that, regardless of the inorganic loading, there is no difference in the immediate or long-term antimicrobial physical and chemical properties of the experimental composites.

## 2. Materials and Methods

**Figure 1:**
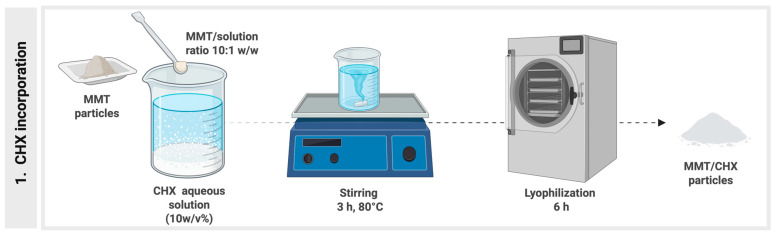
Schematic illustration of the CHX incorporation into MMT particles. Created in BioRender. Boaro, L. (2025) https://BioRender.com/m45v255 (Agreement number # SV28P2PLQS).

Figure 1 presents a schematic representation of the incorporation of CHX into montmorillonite (MMT) particles, as described before by Boaro et al. [2].

**Figure 2:**
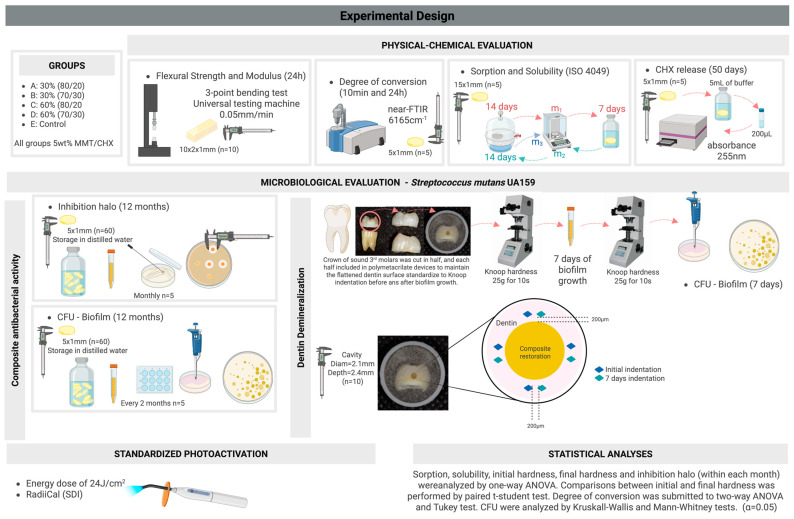
Experimental design–schematic illustration to summarize all experiments completed in the present study. Figure Created in BioRender. Boaro, L. (2025) https://BioRender.com/2a392hg (Agreement number # SR28P2OM9Y).

Additionally, Figure 2 illustrates a concise overview of the experimental design.

### 2.1. Experimental Resin Composites Synthesis

Five experimental resin composites were synthesized with same proportion in weight as 2,2-bis [4-[2-hydroxy-3-methacryloxy) propoxi]phenyl]-propane (BisGMA, ESSTECH Technology, Essington, PA, USA) and triethylene glycol dimethacrylate (ESSTECH). The photo-initiator system was composed by 0.8 wt% camphorquinone (Sigma Aldrich, São Paulo, Brazil) and 1 wt% 2-(dimethylamino)ethyl methacrylate (DMAEMA, Sigma Aldrich). In addition, 5 wt% montmorillonite organophilized (Cloisite 30B^®^, Nanoclay Powder, Nanochemazone, Leduc, AB, Canada) and loaded with CHX was added to the resin matrix. Silanized barium glass and colloidal silica were added in total concentrations of 30 and 60 wt%, respectively, in the proportions 80:20 or 70:30 using a Speed Mixer at 3500 rpm for 3 min (SpeedMixer DAC 150 FVZ, Hauschild, Farmington Hills, MI, USA). A control group without inorganic filler and with 5 wt% montmorillonite loaded with CHX was also included, as presented on Table 1. The photopolymerization was standardized for all tests with an energy dose of 24 J/cm^2^ (1200 mW per 20s), performed using a LED device (Radii Cal, SDI, Bayswater, Australia).

### 2.2. Physical–Chemical Evaluation of the Experimental Composites

#### 2.2.1. Degree of Conversion

**Figure 3:**
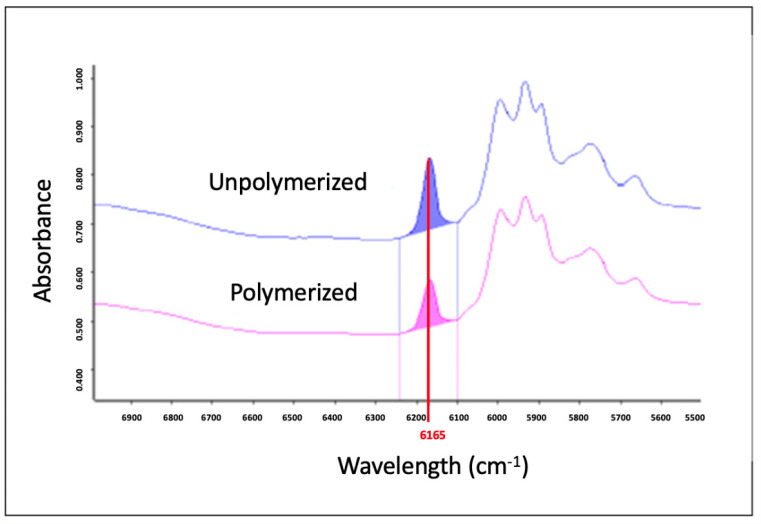
Spectrum of resin composite unpolymerized and polymerized with the area under the 6165 cm^−1^ peak determined.

Circular samples were made with a 5 mm diameter and 1 mm height using a silicon mold (n = 5). The degree of conversion was measured at 10 min and 24 h after photoactivation by near Fourier-transform infrared spectrometry (FTIR, Vertex 70, Bruker Optik GmbH, Ettlingen, Germany), with the resolution at 6 cm^−1^. In the software Opus v.6 (Bruker Optics), the area under the vinyl peak at 6165 cm^−1^ was measured before and after photoactivation to calculate the conversion of the material each time (Figure 3). The composite specimens were photoactivated as described above.

#### 2.2.2. Sorption and Solubility

The sorption and solubility assays were performed according to the ISO 4049 [11]. Samples were made with a 15 mm diameter and 1 mm height, using a steel mold (n = 5) and photoactivated on both sides with a LED unit (Radii Cal, SDI, Bayswater, Australia), with an energy dose of 24 J/cm^2^. The samples size was measured using a digital caliper to calculate the sample volume. The samples were dried in a vacuum desiccator at 37 °C for 14 days and weighed in analytical balance to obtain the m_1_ measurement. Then, the samples were immersed in distillated water at 37 °C for 7 days, when they were weighed to obtain the m_2_ measurement. The samples were dried in a vacuum desiccator at 37 °C for 14 days again and weighed to obtain the m_3_ measurement. The sorption and solubility of the materials were calculated according to Equations (1) and (2), respectively, below, where SR is sorption, SL is solubility, m_1_ is the mass (µg) obtained after initial drying, m_2_ is the mass (µg) obtained after immersion in water, m_3_ is the final mass (µg) after final drying of the samples, and V is the sample volume (mm^3^):

(1) $$SR=(\frac{m2-m1)}{V})$$

(2) $$SL=(\frac{\left(m1-m3\right)}{V})$$

#### 2.2.3. Flexural Strength and Elastic Modulus

Specimens’ bars with 10 × 2 × 1 mm^3^ (n = 10) were made using a stain mold and photopolymerized as described previously. The samples were stored in distillated water at 37 °C and tested 24 h after photoactivation in three bending tests using a universal test machine (Instron) with a span of 8 mm and speed of 0.5 mm/min.

#### 2.2.4. Chlorhexidine Release

Specimens were made using a silicone matrix with a 5 mm diameter and 1 mm thickness (n = 5), photoactivated as described previously. The samples were weighted, immersed in 5 mL saline buffer (pH 7, 50 mM), and maintained at 37 °C in a shaker with mechanical agitation of 80 rpm. Aliquots of 200 μL were removed at 0, 1, 2, 3, and 4 h every 24 h for 5 days and weekly for 50 days. CHX was quantified by analysis of the absorbance at 255 nm [12], obtained using a spectrophotometer for microplates (i3x Spectramax, Molecular Devices, San Jose, CA, USA).

### 2.3. Microbiological Evaluations

#### 2.3.1. Inhibition Halo (12 Months)

Samples with a 5 mm diameter and 1 mm height were prepared using a silicone mold and light cured as described previously. Sixty samples were made, and the samples were immersed in distillated water at 37 °C until use, with the water replaced weekly.

A bacterial suspension of *Streptococcus mutans* UA159 was adjusted in the spectrophotometer with an absorbance of 0.1 at 660 nm, which is equivalent to 1–2 × 10^8^ CFU/mL. A sterile swab was immersed in this solution and sprayed on a BHI culture plate. Five samples of each material (n = 5) were placed over the plate and incubated in an anaerobiosis generator at 37 °C for 24 h. The inhibition halo was measured using a digital caliper in two perpendicular directions. The mean of these measurements was considered the inhibition halo diameter. The sample diameters were also measured so small variations in the sample diameters did not affect the results. The experiment was repeated monthly for 12 months, testing the samples previously stored in distillated water.

#### 2.3.2. CFU–Biofilm Assay (12 Months)

Samples with a 5 mm diameter and 1 mm height were prepared using a silicone mold and light cured as described previously. Thirty-five samples were created, and the samples were immersed in distillated water at 37 °C until use, with the water replaced weekly.

The biofilm growth using a bacterial suspension of *Streptococcus mutans* UA159 was adjusted in the spectrophotometer with an absorbance of 0.1 at 660 nm, which is equivalent to 1–2 × 10^8^ CFU/mL. In addition, 120 µL/well of bacterial inoculum were added to 1.5 mL of culture medium composed by BHI broth and 20% sacarose using a 24-well plate, and the samples were immersed individually in the wells and incubated in an oven with an anaerobic environment for 48 h. The medium was replaced every 24 h, without a new addition of bacterial inoculum. After the culture time, the samples were immersed in saline solution to remove weak attached bacteria, transferred to a new tube to be vortexed, sonicated, and diluted from 10^2^.

The solution was spread over an agar plate using a Drigalski strap around the flames of the Bulsen burner for each sample. The plates were incubated in an anaerobiosis jar for 48 h, when the colonies were counted by eyesight (CFU/mL). The experiment was performed initially (0 month) and then repeated bimonthly for 12 months, testing the samples previously stored in distillated water as described above (n = 5 twice a month).

#### 2.3.3. Dentin Demineralization

The project was approved by the Research Ethics Committee with Human Subjects of Santo Amaro University, and informed consent from participants that donated an extracted tooth for the study was obtaining and signed. Fifty samples (n = 10 per group) were obtained, cutting twenty-five extracted human third molars at the enamel–cement junction and then in the buccolingual direction, resulting in two faces (mesial and distal), which were slightly planed. The specimens were fixed on a glass plate, using a double-sided tape, and included with self-polymerizing acrylic resin (VIPI, Pirassununga, São Paulo, Brazil) in PVC tubes (d = 16 mm, h = 8 mm), with the plane surface exposed. The enamel faces were flattened with 600 and 1200 grit water sandpapers (Norton, Guarulhos, São Paulo, Brazil) under cooling using a polisher (PVV, TECLAGO, Itapevi, São Paulo, Brazil). Circular cavities (d = 2.1 mm and h = 2.4) were prepared in the central portion of the planed faces under constant cooling with a FG 2131 diamond tip (MICRODONT, São Paulo, SP, Brazil). The specimens were randomly assigned to the 5 experimental groups. The cavity walls were conditioned with 37% phosphoric acid (Condac, FGM, Joinville, Santa Catarina, Brazil) for 15 s and then washed for 15 s with a triple syringe. Excess water was removed with air jets, and the single-bottle adhesive system (3M™ Adper™ Single Bond 2, MN, USA) was actively applied using a microbrush, then light-cured as described above. Finally, the cavities were restored in a single increment with one of the composites and light-curing as standardized for all experiments.

In order to evaluate the demineralization process in vitro, the initial surface hardness (KHN_i_) was measured 24 h after restoration, and the samples were randomly allocated into the five groups (n = 10).

The samples were submitted to the exact same protocol of biofilm growth described in Section 2.3.2 and incubated individually in a 12-well plate with 3 mL of culture medium containing *Streptococcus mutans* UA159 for 7 days to promote demineralization by biofilm presence and obtain the number of CFU/mL.

After 7 days of biofilm growth, the final surface hardness (KHN_f_) was measured again and the degree of demineralization indirectly determined by each restorative condition. The hardness was measured using the Microhardness Tester (Shimadzu HMV 2T, Kyoto, Japan), equipped with a Knoop-type diamond pyramid indenter, applying a 25 g load for 10 s. Initial and final hardness were measured at 4 standardized points around the restoration, approximately 200 μm from its edge close to the initial ones but not placed on the exact same spots. The percentage of difference between them (ΔKHN) was calculated according to Equation (3):

(3) $$∆KHN=\frac{({KHN}_{i}-{KHN}_{F})\times 100}{{KHN}_{i}}$$

As described in Section 2.3.2, after the culture time, the samples (restored dentin) were immersed in saline solution to remove weakly attached bacteria and transferred to a new tube to be vortexed, sonicated, and diluted from 10^2^. The solution was spread over an agar plate using a Drigalski strap around the flames of the Bulsen burner for each sample. The plates were incubated in an anaerobiosis jar for 48 h, when the colonies were counted by eyesight (CFU/mL).

### 2.4. Statistical Analysis

**Figure 4:**
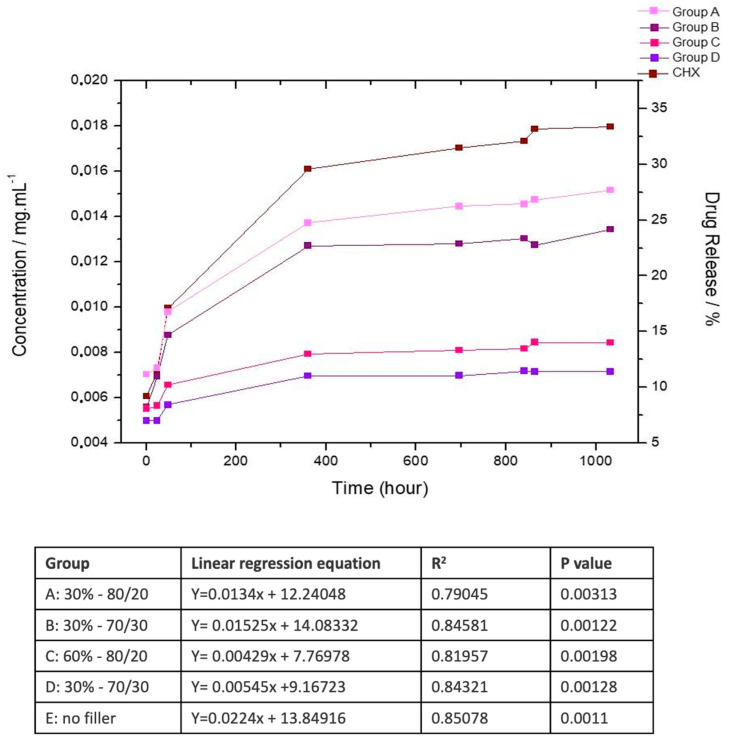
Chlorhexidine (CHX) release over 50 days (n = 5) expressed as concentration (mg/mL, left *y*-axis) and cumulative percentage release (right *y*-axis). Group A: 30–80/20; Group B: 30–70/30; Group C: 60–80/20; Group D: 60–70/30; Group E: control-only bioactive particles, no reinforcement filler. The linear regression equations, coefficients of determination (R^2^), and *p*-values for each group are presented in the accompanying table, indicating a strong model fit during the early release phase.

Data were submitted to normality and homoscedastic testing. Sorption, solubility, initial hardness, final hardness, and inhibition halo (within each month) were analyzed by one-way ANOVA. Comparisons between initial and final hardness were performed by paired *t*-student tests. Degree of conversion was submitted to two-way ANOVA and Tukey’s test. CFU were analyzed by Kruskall–Wallis and Mann–Whitney tests. The global level of significance of 95% (α = 0.05) was adopted in all the statistical tests. The release data for each group were fitted using a linear regression model over the 50-day evaluation period, corresponding to the most stable and linear portion of the release profile. This analysis allowed the determination of the initial release rates and assessment of the influence of the filler content and ratio. The regression equations, coefficients of determination (R^2^), and *p*-values for each group are presented in the accompanying table of Figure 4, indicating a strong model fit during the early release phase.

## 3. Results

The results of the physicochemical evaluation of the experimental composites are presented in Table 2. The degree of conversion did not differ among the experimental groups, nor at 10 min at 24 h. For all the materials, the conversion increases from 10 min to 24 h (Table 2).

Also, the sorption and solubility of the experimental composites were statically similar independent from the filler concentration and barium glass and silica proportions (Table 2).

The flexural strength and elastic modulus at 24 h were highest in the group with 60 wt% filler independent from the barium glass and silica ratio and lowest in the control group without a filler concentration (Table 2).

The CHX released from the experimental composites shows an accentuated increase until around 15 days, when the release reaches a plateau, and the release occurs slowly until 50 days (Figure 4). The materials with higher filler concentration (60%) present lower release of the CHX over time. All experimental groups exhibited an initial increase in CHX release, reaching a plateau before the end of the 50-day evaluation. Linear regression analysis of the release data demonstrated strong correlations (R^2^ = 0.79–0.85, *p* < 0.005) for all groups, confirming the linearity of the early release phase. Composites with a lower filler content (Groups A, B, and control) showed higher release rates, while those with 60% filler (Groups C and D) exhibited slower release profiles.

The halo inhibition assay of the specimens stored in distilled water for periods ranging from 1 month to 1 year shows a prolonged antimicrobial effect of the materials containing MMT/CHX compared to the control. The control exhibited halo inhibition up to the fourth month, while the experimental materials inhibited bacterial growth between 9 and 12 months (Table 3 and Figure S1). The material with 60% loading in a 70:30 ratio of barium glass to silica was the only one that demonstrated halo inhibition throughout the 12-month evaluation period. At a 30% loading concentration, the same 70:30 ratio exhibited antimicrobial effects up to the 11th month. In contrast, the 80:20 ratio of barium glass to silica showed antimicrobial effects until the 9th and 10th months for final concentrations of 60% and 30%, respectively.

The formation of biofilm on composite resin specimens stored for 1–12 months indicates that the control group had an equal or lower number of colony-forming units (CFUs) compared to the experimental groups up to the 8th month. However, from the 8th to the 12th month, the materials with 60% loading in both ratios of barium glass to silica demonstrated greater biofilm inhibition, with fewer CFUs than the control group (Table 4).

The initial hardness data of the dentin indicate no significant differences among the groups at the baseline, as determined by the allocation of the teeth (Table 5 and Figure S2). Seven days after exposure to the biofilm, no differences were observed between the groups, suggesting that, regardless of the restorative material used, all dental fragments underwent demineralization to the same extent due to the presence of the biofilm. The analysis of CFUs obtained from the biofilm formed on restored tooth specimens indicates that the materials with 60% loading, in both barium glass-to-silica ratios (80:20 and 70:30), exhibited fewer CFUs than the control and the materials with 30% loading, indicating a greater antimicrobial effect of these materials with higher loading concentrations (Table 5).

## 4. Discussion

In this study, we could demonstrate, for the first time, the long-term release over 12 months of CHX in experimental dental composites and its antimicrobial effect in the immediate and long-term using halo inhibition and biofilm assays, depending on the filler load. Therefore, the null hypothesis was partially denied, since differences were observed in the dependence of filler concentration regarding antimicrobial activity, flexural strength, and elastic modulus, but the conversion and solubility were kept similar in the composites.

The filler concentration and type have been shown to affect the physical–chemical properties of composites, such as the flexural strength, elastic modulus, sorption, and degree of conversion of the experimental composites [13,14,15,16]. However, in the present study, only the flexural strength and elastic modulus increased when the total filler increased. Absence of an effect in the degree of conversion could be due to the filler size being higher than the light wavelength [16] not affecting the light scattering, as a similar effect has been observed in other studies [14,17]. The sorption and solubility were according to the specifications of ISO 4049, and although a lower sorption and solubility could be expected with the increase in filler amount [18,19], apparently, the similar polymeric matrix and similar conversion were decisive in determining these results [20], which were similar among the groups.

In this way, the similarity in conversion, sorption, and solubility indicates a similarity in the polymeric matrix structure and residual monomers; therefore, we concentrated on the influence of the filler amount and types on the CHX release and on the antimicrobial performance observed and not on the polymeric structure variables.

Although the release of CHX is significant up to the 15th day, after which it reaches a plateau, the results demonstrate a prolonged antimicrobial action of chlorhexidine. This is evident in both the microbial inhibition halo assay and the reduction of biofilm CFUs assay, highlighting a low minimum inhibitory concentration for this drug, as previously described in the literature [21]. Indeed, the release of CHX in resin composites was observed for 16–28 days [6,22]. When associated with mesoporous silica carrier particles or other fillers or bioactive particles, this release was higher and more gradual, taking longer to reach a plateau [6,22]. In the absence of carrier particles, there is an excessive and uncontrolled release within the first few hours [6]. In the present study, besides the carrier particles, the presence of filler particles in the restorative composite also affected the CHX release rate. In resins without fillers, about 35% of the CHX was released over 50 days. However, in materials with 30% reinforcing particles, this rate dropped to 25–30%, and in materials with 60% filler particles, it further decreased to 15–20%. It has been shown that the water uptake in resin composites with 2 mm thickness can take around 60 days, depending on the composition [23], which would explain the different cycles of drug release and antimicrobial effect in biofilm growth, as observed in Table 4 and Figure S2 of the Supplementary Materials. Therefore, the more superficial MMT/CHX particles would be responsible for the short-time CHX release, whereas the long-term release would depend on the water uptake in the core of the material, which is guided by the degree of hydrophobicity of the polymeric matrix and by the degradation rate [24]. In fact, a study added 2% CHX in nanohybrid and ormocer composites and observed a CHX release for 87 days in the ormocer composite and only 14 days in the nanohybrid material [25], showing the importance of composition to drug release. Also, the use of a carrier affects the rate of CHX release [26]. However, the CHX release profile was not able to be measured farther than 50 days, as the elution of the residual monomers and degradation products interfered in the spectrum, making the measurement of CHX unfeasible.

The antimicrobial inhibition halo assay showed some antimicrobial effects on the control group up to the fourth month. In contrast, in the groups with inorganic fillers, the effect extended to 9–12 months, depending on the experimental group. This increase in duration of antimicrobial activity was attributed to the presence of inorganic fillers in the composites, which decreased the water uptake. Materials with a 70/30 ratio of barium glass/silica exhibited a more prolonged effect (12 and 11 months) compared to those with an 80/20 ratio (9 and 10 months) for the 60% and 30% concentrations, respectively. The greater packing density of the filler particles observed in the 70/30 ratio due to the difference in average particle sizes likely reduced the spacing between them, hindering the diffusion of CHX and, consequently, prolonging the antimicrobial effect [27]. Furthermore, the larger surface area of the filler particles promotes hydrolysis at the filler/matrix interface, facilitating greater CHX release [20].

In a previous study by our research group, antibacterial activity was shown to depend on the concentration of the MMT/CHX complex. The inhibition zone in agar was influenced by the presence of CHX but not by the presence of MMT alone [2], indicating that the observed antimicrobial effect was solely due to CHX. In short-term studies, MMT/CHX particles were able to inhibit halo formation in cultures of *S. mutans*, *S. aureus*, *E. faecalis*, and *P. gingivalis* when incorporated into composite resins, acrylic resins, or endodontic cements [2,28]. On the other hand, CHX without carrier particles, when incorporated into resin cements, effectively inhibited bacterial halos against *S. mutans*, *L. casei*, *A. naeslundii*, and *E. faecalis* [29].

In none of the groups did the diameter of the inhibition halo show a linear relationship with the analysis time. However, in this type of assay, the halo size depends not only on the drug release from the material but also on its ability to diffuse in the agar medium. Therefore, it is considered relevant to assess only the presence or absence of inhibitory effects, making this assay not a reliable indicator for quantifying antimicrobial effects [30,31]. For such analysis, colony-forming units were quantified in bacterial biofilm growth assays.

The analysis of colony-forming units (CFUs) in biofilms on the surface of resin composite specimens indicates that the lowest biofilm formation occurred, on average, for all materials around the sixth to eighth month. Beyond this period, there was a significant increase in CFUs in the control group and in materials with 30% filler particles, whereas, in materials with 60% filler, this increase was less pronounced, regardless of the ratio between barium glass and silica. Possibly, in materials with a higher inorganic concentration, the slower release of CHX maintains its effect on biofilm formation for a longer period. Although some studies have indicated a gradual release of CHX [2,6,26], and in the literature, just one long-term study on the antibacterial effect of CHX was found, limited to 1 month [26], confirming effective inhibition of *S. mutans* biofilms when carried in mesoporous silica and added to resin composites, which agrees with the findings of the present study. Short-term studies have also demonstrated effective biofilm inhibition by CHX against *S. mutans* [2,6,22].

The number of colony-forming units (CFUs) of biofilm formed on the surface of teeth restored with experimental composites after 7 days was lower in materials containing 60% filler particles, regardless of their ratio, although a higher release of CHX was expected in materials with 0% or 30% filler particles within 7 days, which would result in greater antimicrobial effects. These results can be explained by two factors: (1) A higher percentage of polymeric matrix is more easily degraded in the presence of water and biofilm, creating a more porous surface that facilitates biofilm accumulation and growth; (2) drug release leaves pores on the resin surface that facilitate biofilm accumulation; in materials with higher release, these pores would be larger and therefore more prone to degradation. Additionally, the minimum inhibitory concentration of CHX has been reported as 0.25–1.0 µg/mL for *S. mutans* [21], indicating that the concentration released by composites with a higher filler content was sufficient to achieve antimicrobial reduction, without requiring higher doses.

Despite the differences in biofilm formation, the reduction in surface hardness of dentin adjacent to restorations before and after exposure to biofilm for 7 days was similar across all composite groups. It is believed that this simulated biofilm formation on dental structures was highly aggressive, as, in the oral cavity, biofilm is unlikely to persist for 7 days without mechanical disruption from tooth brushing or by chewing. Furthermore, the specimen configuration, with the presence of acrylic resin, also favored biofilm accumulation and intensified its effect. New specimen designs may potentially be more favorable in identifying differences in hardness variations among experimental groups, although differences in biofilm formation were readily identifiable.

There is a worldwide concern regarding the rational use of antibiotics and, more secondarily, the use of substances with an antiseptic effect, such as CHX [32]. It is still not proven that CHX may lead to bacterial resistance [32], but it cannot be completely discarded considering the increasing use, especially as chlorhexidine gluconate solutions [33]. Therefore, with a safe and adequate use of CHX, it is believed that the risk of promoting the selection of resistant bacteria is reduced [33], which greatest concern is related to exposure for prolonged periods at higher concentrations, such as in the daily use of mouthwashes and toothpaste, for example. Thus, based on the low amount of CHX released in these materials studied, it is believed that there is a low risk of substantially affecting the oral microbiota or even promoting selective pressure for resistant microorganisms. However, more studies should be completed in this way before clinical applications.

In addition, the antimicrobial composites should be addressed as a complementary treatment in order to reestablish the buccal health of the patients, as the addition of MMT/CHX particles has been promising in the long-term reduction of *S. mutans* biofilm. However, this potential could probably be increased by associating it with other particles that have also presented long-term antimicrobial effects, such as polyethyleneimine [34] or mesoporous silica loaded with essential oils [35], or associating them with other antimicrobial strategies such as the inhibition of biofilm adhesion by superhydrophobic surfaces [36] or the use of photocatalytic nanoparticles on the material’s surface [37]. More future studies are needed in order to evaluate these potentialities and the synergic effects among them and also the mechanical properties maintenance for the design of innovative antimicrobial dental resin composites.

## 5. Conclusions

Within the limitations of this study, we can conclude that resin composites with higher amounts of inorganic filler (60wt%), preferentially in a ratio of 70% barium glass and 30% silica associated with 5 wt% MMT/CHX particles, not only increases the flexural strength and elastic modulus but also promotes a more controlled release of chlorhexidine and a long-term antibacterial effect, as evidenced by prolonged inhibition halos and CFU counting in biofilm growth over the specimens’ disc and in restored teeth.

## Figures and Tables

**Table 1:** Groups–experimental composites compositions.

Group	Inorganic Filler		Montmorillonite Loaded with Chlorhexidine (wt%)	Organic Matrix (wt%)	
	Total Concentration (wt%)	Barium Glass/Silica Rate		Monomers	Photo-initiator System
A	30	80/20	5	31.6 BisGMA 31.6 TEGDMA	0.8 camphorquinone 1.0 DMAEMA
B		70/30			
C	60	80/20		16.6 BisGMA 16.6 TEGDMA	
D		70/30			
E	None			46.6 BisGMA 46.6 TEGDMA	

**Table 2:** Physical–chemical properties of the experimental composites. Mean and standard deviation of sorption (mg/mL), solubility (mg/mL), degree of conversion (%), flexural strength (MPa), and elastic modulus (GPa) for the filler concentration and filler ratio. Sorption, solubility, and degree of conversion do not show statistical difference among the groups. Similar letters indicate the absence of statistical difference for flexural strength and elastic modulus.

Groups	Barium Glass/Silica		Sorption (mg/mL)	Solubility (mg/mL)	Degree of Conversion (%)		Flexural Strength (MPa)	Elastic Modulus (GPa)
	Concentration (wt%)	Ratio			10 min	24 h		
A	30	80/20	0.003 (0.002)	0.046 (0.021)	49 (6)	65 (5)	27.4 (6.3) ^BC^	4.0 (1.2) ^B^
B		70/30	0.001 (0.001)	0.039 (0.014)	54 (10)	68 (11)	30.7 (5.4) ^B^	4.0 (0.8) ^B^
C	60	80/20	0.002 (0.004)	0.037 (0.003)	47 (6)	60 (5)	44.6 (12.2) ^A^	9.4 (2.4) ^A^
D		70/30	0.001 (0.001)	0.064 (0.033)	50 (5)	57 (9)	42.7 (9.5) ^A^	9.1 (2.1) ^A^
E	Control *		0.001 (0.001)	0.044 (0.018)	46 (6)	52 (9)	18.9 (5.3) ^C^	3.9 (1.2) ^B^

* Control: group with 5% of MMT/CHX without inorganic filler.

**Table 3:** Inhibition halo of experimental composite specimens. Means (standard deviation) in mm of results over twelve months. In the same column, values followed by the same letter are statistically similar (*p* > 0.05). One-way ANOVA within each month.

Groups	Barium Glass/Silica		Month											
	Concentration (wt%)	Ratio	1	2	3	4	5	6	7	8	9	10	11	12
A	30	80/20	5.9 (1.7) ^AB^	4.5 (1.2) ^A^	1.6 (0.5) ^B^	2.1 (0.7) ^A^	3.1 (0.8) ^A^	5.2 (1.7) ^A^	3.9 (0.6) ^A^	3.8 (0.9) ^A^	1.9 (0.6) ^AB^	4.5 (1.8) ^A^	0.0 (0.0) ^B^	0.0 (0.0) ^B^
B		70/30	10.1 (1.7) ^A^	10.1 (1.7) ^A^	3.2 (0.4) ^A^	1.6 (0.5) ^A^	0.0 (0.0) ^B^	2.1 (0.6) ^B^	0.0 (0.0) ^C^	4.8 (0.8) ^A^	3.1 (0.7) ^AB^	3.4 (1.3) ^A^	4.6 (1.2) ^A^	0.0 (0.0) ^B^
C	60	80/20	6.3 (1.3) ^AB^	4.3 (1.3) ^A^	1.6 (0.6) ^B^	2.7 (0.8) ^A^	3.2 (1.0) ^A^	1.3 (0.4) ^B^	2.6 (0.9) ^A^	3.8 (1.1) ^A^	0.9 (0.3) ^B^	0.0 (0.0) ^B^	0.0 (0.0) ^B^	0.0 (0.0) ^B^
D		70/30	12.4 (2.5) ^A^	4.6 (0.8) ^A^	2.1 (0.2) ^A^	1.5 (0.3) ^A^	3.7 (1.1) ^A^	2.5 (0.8) ^B^	1.7 (0.5) ^B^	3.11 (1.1) ^A^	3.6 (1.1) ^A^	4.6 (1.2) ^A^	1.3 (0.3) ^A^	2.8 (1.0) ^A^
E	Control *		3.8 (1.0) ^B^	6.8 (1.4) ^A^	0.0 (0.0) ^C^	1.7 (0.5) ^A^	0.0 (0.0) ^B^	0.0 (0.0) ^C^	0.0 (0.0) ^C^	0.0 (0.0) ^B^	0.0 (0.0) ^C^	0.0 (0.0) ^B^	0.0 (0.0) ^B^	0.0 (0.0) ^B^

* Control: group with 5% of MMT/CHX without inorganic filler.

**Table 4:** Biofilm on experimental composite specimens. Means (standard deviation) of colony-forming units (CFU/mL) counted at a dilution of 10^2^. In the same column, values followed by the same letter are statistically similar (*p* > 0.05). One-way ANOVA within each month.

Groups	Barium Glass/Silica		0 Month	2nd Month	4th Month	6th Month	8th Month	10th Month	12th Month
	Concentration (wt%)	Ratio							
A	30	80/20	0	2.3 (1.7) ^A^	23.3 (1.4) ^A^	1.5 (1.3) ^AB^	1.8 (2.4) ^A^	12.8 (2.5) ^C^	20.8 (5.3) ^B^
B		70/30	0	0.5 (0.6) ^A^	27.0 (3.6) ^A^	1.5 (1.7) ^AB^	0.8 (1.0) ^A^	51.8 (4.9) ^A^	61.7 (7.8) ^A^
C	60	80/20	0	0.0 (0.0) ^B^	5.8 (1.7) ^B^	0.3 (0.5) ^B^	0.5 (0.6) ^A^	6.0 (1.3) ^C^	7.1 (1.9) ^C^
D		70/30	0	0.0 (0.0) ^B^	9.0 (1.3) ^B^	4.5 (1.2) ^A^	0.8 (1.0) ^A^	4.3 (1.3) ^C^	5.6 (1.8) ^C^
E	Control *		0	0.0 (0.0) ^B^	14.8 (3.7) ^AB^	0.0 (0.0) ^B^	0.0 (0.0) ^A^	24.5 (3.8) ^B^	40.5 (5.7) ^AB^

* Control: group with 5% of MMT/CHX without inorganic filler.

**Table 5:** Demineralization in dentin specimens. Means (standard deviations) for Knoop microhardness data in dentin, percentage reduction in microhardness from the initial day and after 7 days of biofilm growth, and colony-forming units (CFU/mL) counted at a dilution of 10^2^. Values within the same column followed by the same uppercase letter are statistically similar (*p* > 0.05). Values within the same line followed by the same lower case letter are statistically similar (*p* > 0.05).

Groups	Barium Glass/Silica		Knoop Microhardness			UFC 7 Days
	Concentration wt%	Ratio	Initial	7 Days	Reduction (%)	
A	30	80/20	82.9 (19.4) ^Aa^	41.6 (18.7) ^Ab^	49	19.0 (3.8) ^A^
B		70/30	72.5 (16.8) ^Aa^	37.2 (14.3) ^Ab^	49	18.4 (2.4) ^A^
C	60	80/20	76.45 (20.1) ^Aa^	44.21 (16.9) ^Ab^	42	6.2 (1.3) ^B^
D		70/30	91.8 (21.2) ^Aa^	56.6 (13.9) ^Ab^	38	12.0 (1.9) ^B^
E	Control *		84.1 (19.5) ^Aa^	38.5 (18.4) ^Ab^	54	22.0 (1.8) ^A^

* Control: group with 5% of MMT/CHX without inorganic filler.

## Data Availability

The original contributions presented in this study are included in the article/Supplementary Material. Further inquiries can be directed to the corresponding author.

## Supplementary Materials

The following supporting information can be downloaded at https://www.mdpi.com/article/10.3390/pharmaceutics17091144/s1: Figure S1: Mean and standard deviation (mm) of Inhibition halo on experimental composite over twelve months. Figure S2: Mean and standard deviation of colony-forming units (CFU/mL) counted at a dilution of 10^2^, from Biofilm growth on experimental composite specimens.

## References

[1] Varghese E.J., Sihivahanan D., Venkatesh K.V. (2022). Development of Novel Antimicrobial Dental Composite Resin with Nano Cerium Oxide Fillers. Int. J. Biomater..

[2] Boaro L.C., Campos L.M., Varca G.H., Dos Santos T.M., Marques P.A., Sugii M.M., Saldanha N.R., Cogo-Müller K., Brandt W.C., Braga R.R. (2019). Antibacterial resin-based composite containing chlorhexidine for dental applications. Dent. Mater..

[3] Kikuchi L.N., Freitas S.R., Amorim A.F., Delechiave G., Catalani L.H., Braga R.R., Moreira M.S., Boaro L.C., Goncalves F. (2022). Effects of the crosslinking of chitosan/DCPA particles in the antimicrobial and mechanical properties of dental restorative composites. Dent. Mater..

[4] Poppolo Deus F., Ouanounou A. (2022). Chlorhexidine in Dentistry: Pharmacology, Uses, and Adverse Effects. Int. Dent. J..

[5] Spencer P., Ye Q., Misra A., Goncalves S.E., Laurence J.S. (2014). Proteins, pathogens, and failure at the composite-tooth interface. J. Dent. Res..

[6] Zhang J.F., Wu R., Fan Y., Liao S., Wang Y., Wen Z.T., Xu X. (2014). Antibacterial dental composites with chlorhexidine and mesoporous silica. J. Dent. Res..

[7] Wang X., Du Y., Luo J. (2008). Biopolymer/montmorillonite nanocomposite: Preparation, drug-controlled release property and cytotoxicity. Nanotechnology.

[8] Wu Y., Zhou N., Li W., Gu H., Fan Y., Yuan J. (2013). Long-term and controlled release of chlorhexidine-copper(II) from organically modified montmorillonite (OMMT) nanocomposites. Mater. Sci. Eng. C Mater. Biol. Appl..

[9] Alexandre M., Dubois P. (2000). Polymer-layered silicate nanocomposites: Preparation, properties and uses of a new class of materials. Mater. Sci. Eng. R. Rep..

[10] Campos L.M., Boaro L.C., Santos T.M., Marques P.A., Almeida S.R., Braga R.R., Parra D.F. (2017). Evaluation of flexural modulus, flexural strength and degree of conversion in BISGMA/TEGDMA resin filled with montmorillonite nanoparticles. J. Compos. Mater..

[11] (2019). Dentistry—Polymer-Based Restorative Materials.

[12] Young A.M., Ng P.Y., Gbureck U., Nazhat S.N., Barralet J.E., Hofmann M.P. (2008). Characterization of chlorhexidine-releasing, fast-setting, brushite bone cements. Acta Biomater..

[13] Randolph L.D., Palin W.M., Leloup G., Leprince J.G. (2016). Filler characteristics of modern dental resin composites and their influence on physico-mechanical properties. Dent. Mater..

[14] Gonçalves F., Kawano Y., Braga R.R. (2010). Contraction stress related to composite inorganic content. Dent. Mater..

[15] Halvorson R.H., Erickson R.L., Davidson C.L. (2003). The effect of filler and silane content on conversion of resin-based composite. Dent. Mater..

[16] Garoushi S., Vallittu P.K., Watts D.C., Lassila L.V. (2008). Effect of nanofiller fractions and temperature on polymerization shrinkage on glass fiber reinforced filling material. Dent. Mater..

[17] Atai M., Watts D.C. (2006). A new kinetic model for the photopolymerization shrinkage-strain of dental composites and resin-monomers. Dent. Mater..

[18] Prati C., Mongiorgi R., Bertocchi G., Baldisserotto G. (1991). Dental composite resin porosity and effect on water absorption. Boll. Soc. Ital. Biol. Sper..

[19] Alsharif S., Hazizan M.A., El-Aziz N., Ahmad Z. (2013). Simulated Body Fluid Sorption and Solubility of Silica Reinforced Dental Resin Composites. Adv. Mater. Res..

[20] Santerre J.P., Shajii L., Leung B.W. (2001). Relation of dental composite formulations to their degradation and the release of hydrolyzed polymeric-resin-derived products. Crit. Rev. Oral Biol. Med..

[21] Järvinen H., Tenovuo J., Huovinen P. (1993). In vitro susceptibility of *Streptococcus mutans* to chlorhexidine and six other antimicrobial agents. Antimicrob. Agents Chemother..

[22] Cheng L., Weir M.D., Xu H.H., Kraigsley A.M., Lin N.J., Lin-Gibson S., Zhou X. (2012). Antibacterial and physical properties of calcium-phosphate and calcium-fluoride nanocomposites with chlorhexidine. Dent. Mater..

[23] Shah M.B., Ferracane J.L., Kruzic J.J. (2009). R-curve behavior and toughening mechanisms of resin-based dental composites: Effects of hydration and post-cure heat treatment. Dent. Mater..

[24] Ferracane J.L. (2006). Hygroscopic and hydrolytic effects in dental polymer networks. Dent. Mater..

[25] Berghaus E., Muxkopf G.A., Feddersen S., Eisenburger M., Petersen S. (2022). Antimicrobial agents in dental restorative materials: Effect on long-term drug release and material properties. Eur. J. Oral Sci..

[26] Cao L., Yan J., Luo T., Yan H., Hua F., He H. (2023). Antibacterial and fluorescent clear aligner attachment resin modified with chlorhexidine loaded mesoporous silica nanoparticles and zinc oxide quantum dots. J. Mech. Behav. Biomed. Mater..

[27] Wang R., Habib E., Zhu X.X. (2018). Evaluation of the filler packing structures in dental resin composites: From theory to practice. Dent. Mater..

[28] Gonçalves F., Campos L., Sanches L., Silva L., Santos T., Varca G., Lopes D.P., Cogo-Muller K., Parra D.F., Braga R.R. (2020). Antimicrobial activity and physicochemical performance of a modified endodontic sealer. Res. Soc. Dev..

[29] Mehdawi I.M., Kitagawa R., Kitagawa H., Yamaguchi S., Hirose N., Kohno T., Imazato S. (2022). Incorporation of chlorhexidine in self-adhesive resin cements. Dent. Mater. J..

[30] Bauer A.W., Kirby W.M., Sherris J.C., Turck M. (1966). Antibiotic susceptibility testing by a standardized single disk method. Am. J. Clin. Pathol..

[31] Benkova M., Soukup O., Marek J. (2020). Antimicrobial susceptibility testing: Currently used methods and devices and the near future in clinical practice. J. Appl. Microbiol..

[32] Cieplik F., Jakubovics N.S., Buchalla W., Maisch T., Hellwig E., Al-Ahmad A. (2019). Resistance Toward Chlorhexidine in Oral Bacteria—Is There Cause for Concern?. Front. Microbiol..

[33] Van den Poel B., Saegeman V., Schuermans A. (2022). Increasing usage of chlorhexidine in health care settings: Blessing or curse? A narrative review of the risk of chlorhexidine resistance and the implications for infection prevention and control. Eur. J. Clin. Microbiol. Infect. Dis..

[34] Beyth N., Yudovin-Fearber I., Domb A.J., Weiss E.I. (2010). Long-term antibacterial surface properties of composite resin incorporating polyethyleneimine nanoparticles. Quintessence Int..

[35] Zhong X., Gao F., Wei H., Zhou H., Zhou X. (2021). Functionalization of mesoporous silica as an effective composite carrier for essential oils with improved sustained release behavior and long-term antibacterial performance. Nanotechnology.

[36] Cao D., Zhang Y., Li Y., Shi X., Gong H., Feng D., Guo X., Shi Z., Zhu S., Cui Z. (2017). Fabrication of superhydrophobic coating for preventing microleakage in a dental composite restoration. Mater. Sci. Eng. C Mater. Biol. Appl..

[37] Bryaskova R., Philipova N., Bakov V., Georgiev N. (2025). Innovative Antibacterial Polymer Coatings. Appl. Sci..

