# Supplementary material for: Antibacterial Resin Composites with Sustained Chlorhexidine Release: One-Year In Vitro Study
Source: Pharmaceutics. 2025 Sep 1;17(9):1144. doi: 10.3390/pharmaceutics17091144 (PMC12473463; doi:10.3390/pharmaceutics17091144)
Supplement: Supplementary file 1 [file pharmaceutics-17-01144-s001.zip › pharmaceutics-3763610-supplementary.pdf]

Supplementary Figures

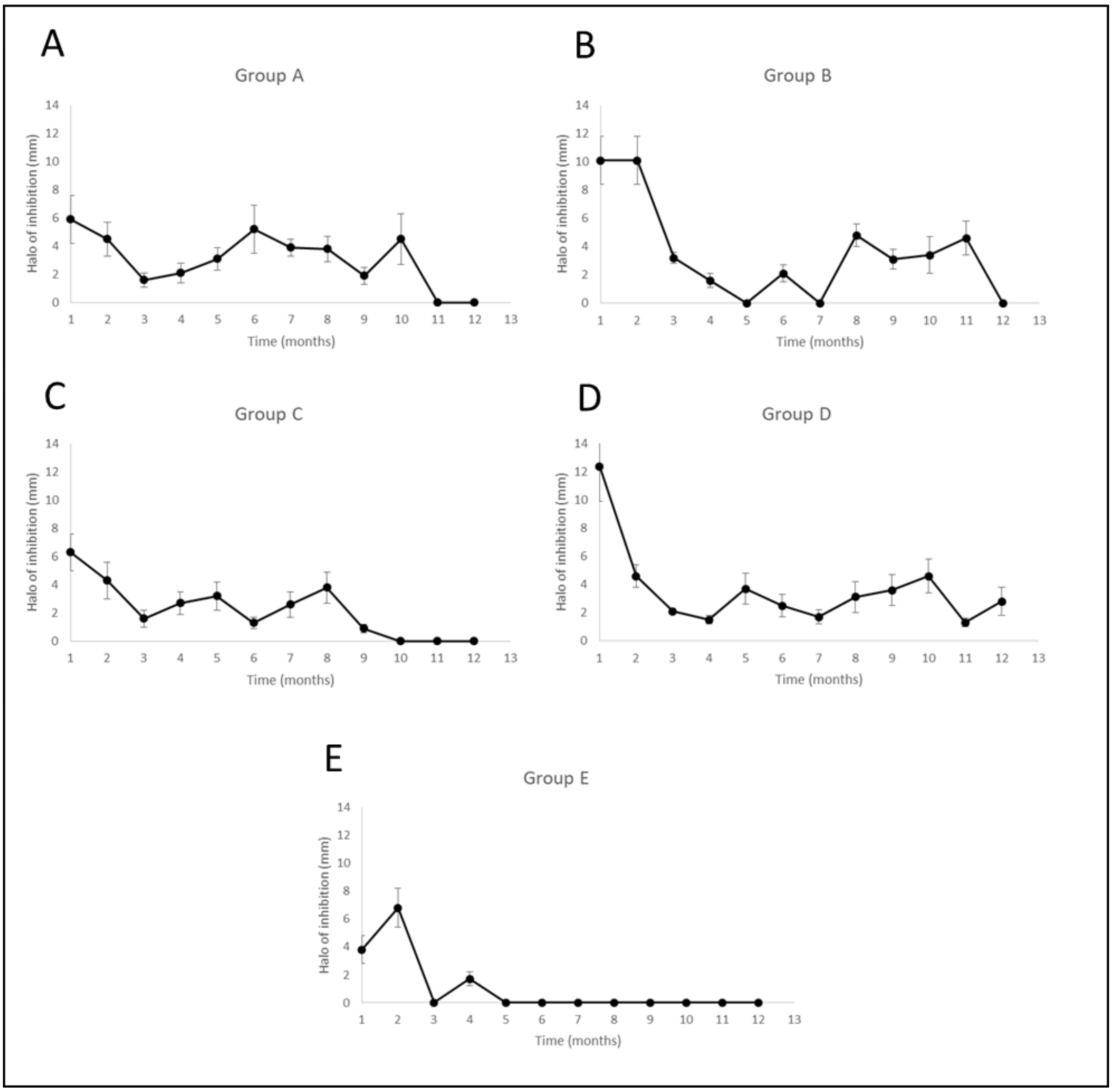

Figure S1: Mean and standard deviation (mm) of Inhibition halo on experimental composite over twelve months.

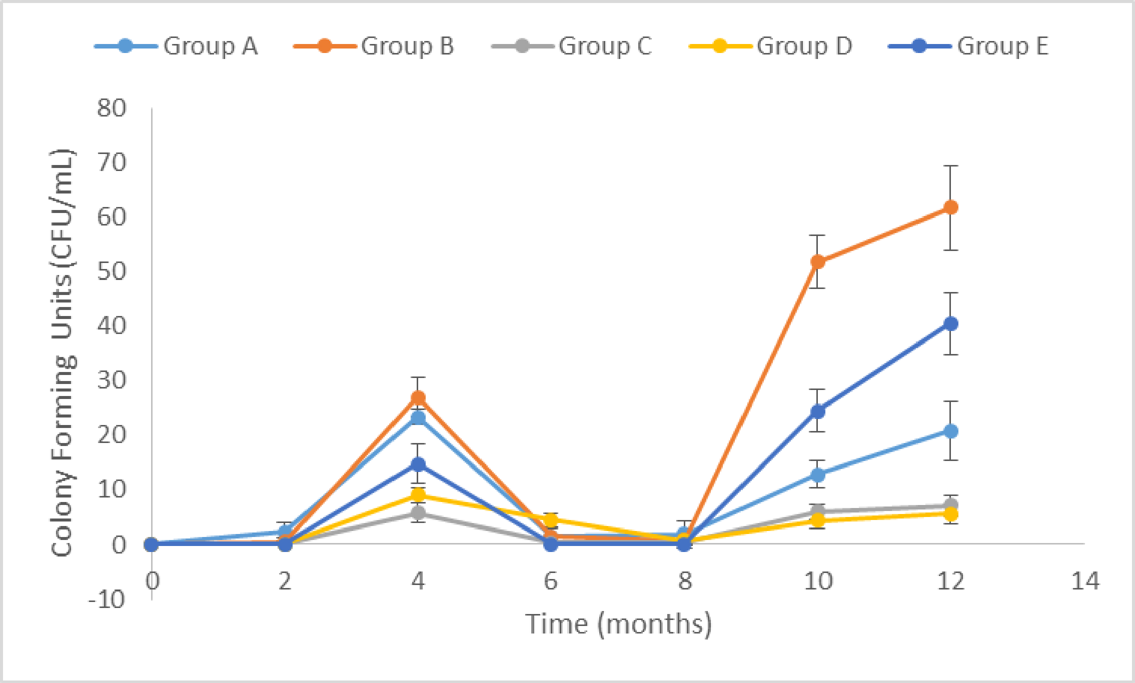

Figure S2: Mean and standard deviation (mm) of colony-forming units (CFU/mL) counted at a dilution of 10^2, from Biofilm growth on experimental composite specimens.
